# Understanding mental health among university students in Kenya: what role do family support and age play?

**DOI:** 10.3389/fpubh.2025.1557058

**Published:** 2025-05-30

**Authors:** Bylhah Mugotitsa, Reinpeter Momanyi, Joseph Kuria, David Amadi, Jacob Masai, Eric Angula, Benjamin Tsofa, Jay Greenfield, Jim Todd, Agnes Kiragga

**Affiliations:** ^1^Data Science Programs, African Population and Health Research Center (APHRC), Nairobi, Kenya; ^2^Strathmore Business School, Strathmore University, Nairobi, Kenya; ^3^Department of Epidemiology, London School of Hygiene and Tropical Medicine, University of London, London, United Kingdom; ^4^Research Department, Medtronics Labs, Nairobi, Kenya; ^5^Health System and Research Ethics Department, Kenya Medical Research Institute (KEMRI), Nairobi, Kenya; ^6^KEMRI-Wellcome Trust Research Programme, Nairobi, Kenya; ^7^Machine Learning & Artificial Intelligence, Committee on Data of the International Science Council (CODATA), Paris, France; ^8^Department of Epidemiology, Catholic University for Health and Allied Sciences, Mwanza, Tanzania

**Keywords:** mental health, depression, anxiety, psychosis, university students, prevalence

## Abstract

**Objective:**

While mental health conditions play a significant role in the global disease burden, their determinants and predictors are still not well understood in Kenya. This study examined the prevalence of mental health conditions among university students and the factors associated with them.

**Methods:**

This cross-sectional study evaluated 1,424 students at Pwani University in Kenya, assessing anxiety, depression, and psychosis, using validated screening tools: the Generalized Anxiety Disorder-7 (GAD-7), Patient Health Questionnaire-9 (PHQ-9), and Psychosis Screening Questionnaire (PSQ). The Chi-square tests analyzed associations, while binary logistic regression identified predictors. Confounders were controlled using multivariable adjustments, with model selection based on both clinical relevance and statistical significance of the variables.

**Results:**

The prevalence of mental health conditions among students was 30.9%. Those from unsupportive families exhibited the highest prevalence at 35.2% (*χ*^2^ = 94.91, *p* < 0.001), while first-year students reported the highest rate among academic levels at 40.7% (*χ*^2^ = 24.38, *p* < 0.001). Students aged 25–29 years were 2.6 times more likely to experience mental health conditions (OR = 2.6, 95% CI: 1.67–3.98, *p* < 0.001). Access to mental health services (*χ*^2^ = 4.62, *p* = 0.032) and mental health insurance (*χ*^2^ = 4.11, *p* = 0.043) were associated with lower odds of mental health conditions, thereby reducing the risk by 34 and 33%, respectively.

**Conclusion:**

The findings highlight the urgent need for age-sensitive, student-centered mental health interventions in Kenyan universities. Specifically, universities should implement targeted support programs for first-year and final-year students who face unique mental health risks due to transitional and graduation-related stressors. Additionally, integrating family engagement initiatives to strengthen family support structures can serve as a protective factor against mental health challenges. Policies aimed at expanding access to mental health insurance and services should also be prioritized. Given the use of non-probabilistic sampling, findings should be interpreted with caution. Future research should investigate longitudinal trends to establish causal relationships and inform the development of evidence-based policies.

## Introduction

Globally, mental health accounts for 13% of the total disease burden, with anxiety and depression being the most common disorders (1). University students face a high risk of mental health conditions due to academic pressures, financial constraints, and social isolation (2, 3). Auerbach et al. reported that up to 35% of university students worldwide experience mental health conditions, yet less than 20% seek professional help (4).

In Africa, mental health conditions remain underrecognized due to stigma, inadequate infrastructure, and limited resources (5). According to the WHO, mental health accounts for 19% of the total disease burden (1). The prevalence rates of depression and anxiety among university students remain high due to a lack of social support, financial instability, and unemployment (2, 6). Studies conducted in Uganda revealed that 30–40% of university students experience symptoms of anxiety and depression (7).

Kenya has recognized mental health as a public health priority (8, 9). In 2021, the Ministry of Health reported that one in every four individuals seeking outpatient services presents with symptoms of a mental health condition (10). University students are at high risk of developing mental health issues, with nearly 40% reporting symptoms of depression or anxiety (11). Limited mental health awareness, financial constraints, and academic stress are the most significant contributors to these alarming statistics (12).

However, despite the growing recognition of mental health challenges, there is limited research on the role of family support and age as determinants of mental health among Kenyan university students. Given the high prevalence of mental health conditions in university students across Kenya and the limited understanding of the contributing factors, this study evaluates the prevalence and determinants of mental health conditions, focusing on family support and age in university students from Kilifi County, Kenya.

## Materials and methods

### Study design and setting

This cross-sectional study was conducted at Pwani University in Kilifi County, Kenya, from September to December 2024. The study population consisted of full-time students enrolled in any year of their studies. A cross-sectional design was selected because it allows for the simultaneous assessment of mental health status and associated factors, making it a practical and efficient approach for identifying prevalence and correlations within a university setting.

### Sampling and sample size calculation

A non-probabilistic purposive sampling method was used to conduct the survey across various academic years. The sample size (n = 1,356) was calculated using the standard formula for estimating a population proportion (13, 14).

#### Sample size formula


$$n=[Z^{2*}\;P^{*}\;(1-P)]/d^{2}$$

Where

*n* = required sample size

Z = Z-score corresponding to the desired confidence level (1.96 for 95%)

P = assumed prevalence (50%)

d = margin of error (3%)

A prevalence of 50% was assumed as it provides the most conservative estimate, maximizes the required sample size, and ensures adequate power to detect associations, as recommended in similar mental health studies (13, 15). A 3% margin of error was determined instead of the standard 5% to increase the precision of the study estimates, given the expected variability in mental health symptoms across different student demographics (16). This narrower margin of error enhances the reliability of prevalence estimates and strengthens the study’s ability to identify meaningful associations (16). While stratified random sampling would have improved representativeness, it was not feasible due to logistical constraints, including limited access to a complete sampling frame and the need to specifically target students with relevant characteristics, such as full-time students and having certain experiences (16). Although purposive sampling allowed for the inclusion of participants with attributes relevant to the study, it introduces potential selection bias and limits generalizability (13, 16). The discussion acknowledges these limitations, emphasizing the need for cautious interpretation of findings and recommending future studies with more representative sampling methods for validation.

### Inclusion and exclusion criteria

The study population consisted of university students from Kilifi County. Eligible participants were full-time students actively enrolled in any year of study at Pwani University, aged 18 years and older, who provided informed consent. Students were excluded if they were studying part-time or were on academic leave, as their academic pressures and social environments might differ from those of full-time students. Additionally, students with a prior clinical diagnosis of severe mental health disorders requiring specialized care were excluded to ensure the study focused on undiagnosed or subclinical mental health symptoms rather than pre-existing conditions. Finally, students who declined to provide informed consent were also excluded in accordance with ethical research principles.

### Data collection and instruments

Data were collected using a structured electronic questionnaire that was pretested among 100 students to assess reliability and validity. The survey covered demographic variables such as age, gender, and year of study, along with socioeconomic factors, including family support, accommodation type, and access to mental health services (12). Behavioral factors such as social media usage and academic stress were also evaluated. Mental health screening was conducted using the PHQ-9 for depression, the GAD-7 for anxiety, and the PSQ for psychosis (17).

Participants were recruited through noticeboards and in-person information sessions held in lecture halls and common areas. To address potential internet accessibility issues, students were encouraged to complete the survey using university Wi-Fi in designated areas such as libraries and computer labs. Research assistants were available to provide support and ensure accessibility for all participants.

Research assistants, trained as data collectors, played a key role in facilitating the study. Although they were not mental health professionals, they received training in ethical considerations, participant confidentiality, and the administration of mental health screening tools. Their responsibilities included guiding students through the informed consent process, assisting with survey completion, and referring participants to university counseling services when necessary. These measures ensured a smooth data collection process while upholding ethical standards.

### Outcome and predictor variables

The primary outcome of this study was the presence of symptoms associated with common mental health conditions, specifically depression and anxiety. Depression was assessed using the Patient Health Questionnaire-9 (PHQ-9), which has a scale ranging from 0 to 27, with scores of 20–27 indicating severe depression. The PHQ-9 comprises nine items, each assessing the frequency of depressive symptoms over the past 2 weeks. The responses are categorized as “not at all,” “several days,” “more than half the days,” and “nearly every day.” Anxiety was evaluated using the Generalized Anxiety Disorder-7 (GAD-7), which has a scale ranging from 0 to 21. The GAD-7 consists of seven items, each assessing the frequency of anxiety symptoms over the past 2 weeks. The responses are similarly categorized as “not at all,” “several days,” “more than half the days,” and “nearly every day. “Both instruments are widely recognized for their reliability and validity across various populations, including university students (17).

### Justification for assessment tools

The PHQ-9 and GAD-7 are brief self-administered tools designed to screen for depression and anxiety, respectively. Their brevity and ease of use make them suitable for large-scale studies, such as those involving university populations. A study conducted among Lithuanian university students demonstrated that both the PHQ-9 and GAD-7 are reliable screening tools for depression and anxiety, with Cronbach’s alpha values of 0.86 and 0.91, respectively (18).

### Cutoff scores and definitions

In this study, a positive outcome for depression was defined as a PHQ-9 score of 10 or higher, indicating moderate to severe depressive symptoms. Similarly, a GAD-7 score of 10 or higher was used to identify moderate to severe anxiety symptoms (19). These cutoff scores are based on established clinical guidelines and have been corroborated by validation studies. For example, in the Lithuanian student sample, a PHQ-9 cutoff of ≥10 resulted in 71% sensitivity and 66% specificity for identifying students at increased risk for mood or anxiety disorders, while a GAD-7 cutoff of ≥9 yielded 73% sensitivity and 70% specificity (18).

### Predictor variables

The study examined various predictor variables to assess their relationship with the presence of mental health conditions among participants:

#### Demographic factors

Age (2, 20), gender (20, 21), and marital status were collected. Age was treated as a continuous variable and divided into groups. Gender options included male and female, while marital status was categorized as single, married, or separated/divorced.

#### Socioeconomic factors

Family Support (2, 21): Assessed using a 5-point Likert scale ranging from very poor (1) to very strong (5), which is later categorized as low (1–2), moderate (3), or high (4–5) (22).

#### Accommodation arrangements

Participants indicated their living situations, such as living with family at home, in a university hostel, or in private rentals (23).

Academic stress (2): This is evaluated using the Perceived Stress Scale (PSS-10), a standardized tool for measuring perceived stress levels. Scores of 14 or higher indicate moderate to high levels of stress.

#### Behavioral factors

Media Use (2) is defined as the number of hours spent daily on social media, streaming platforms, or gaming. Categories include low (<2 h), moderate (2–5 h), and high (>5 h). Research indicates a significant association between problematic social media use and higher mental health scores, suggesting poorer mental health status.

Utilization of Mental Health Services: Participants reported whether they had ever sought counseling or psychiatric services, which was recorded as a binary variable (Yes/No).

### Cultural adaptations

While the PHQ-9 and GAD-7 were initially developed in Western contexts, their applicability in diverse cultural settings has been explored. For instance, a study involving the translation, cultural adaptation, and validation of these tools into Kinyarwanda for refugees and migrants in the United States found that they have good internal consistency, with Cronbach’s alpha values of 0.85 for the PHQ-9 and 0.92 for the GAD-7 (24). Although specific validations in Kenyan university populations are limited, these findings suggest that the PHQ-9 and GAD-7 can be effectively adapted for various cultural contexts.

### Data analysis

Statistical analysis was conducted using STATA version 18.0 (STATA Corporation, College Station, Texas), which facilitated data cleaning, descriptive statistics, and inferential analyses. Descriptive statistics summarized the data, presenting frequencies and percentages for categorical variables, while means and standard deviations were calculated for continuous variables (25).

A bivariate analysis was conducted using the Chi-square test to examine associations between predictor variables and mental health conditions (26). The Chi-square test was preferred over Fisher’s exact test due to the sample size, which satisfied the assumptions for the Chi-square test (26). Univariate binary logistic regression was performed to explore relationships between predictor variables and the presence of mental health conditions (27). This analysis provided crude odds ratios (ORs) with 95% confidence intervals, helping to identify significant factors for further analysis. Logistic regression was chosen due to its suitability for binary outcomes (28).

A multivariate logistic regression analysis was conducted to identify predictors of mental health conditions. Variables were incorporated into the final model based on both clinical relevance and statistical significance from the univariate analysis, using a threshold of *p* of ≤ 0.25 for inclusion (28). This threshold was selected because it allows for a more inclusive selection of variables for multivariable modeling, as recommended in prior literature (28). Clinical relevance was assessed based on previous studies and established risk factors for mental health conditions (2, 3, 7, 20, 21). Examples of clinically relevant variables included age, socioeconomic status, and prior mental health diagnoses.

The assumptions of logistic regression were checked prior to modeling. Multicollinearity was assessed using the Variance Inflation Factor (VIF), with values below 10 indicating acceptable levels (29). The linearity of continuous predictors in log odds was evaluated using the Box-Tidwell test.

The predictive performance of the final model was evaluated using the Receiver Operating Characteristic (ROC) curve (30). The Area Under the Curve (AUC) was 0.7167, indicating an acceptable discriminatory ability to differentiate between students with and without mental health conditions. Additionally, model calibration was assessed using the Hosmer-Lemeshow goodness-of-fit test to ensure the adequacy of the model fit (31).

## Results

### Sociodemographic characteristics

Table 1 shows the demographic characteristics of the participants. A total of 1,424 students participated in the study, with a majority being female (54.6%). The majority of participants were in their first year of study (41.3%), came from supportive families, and reported spending 1–3 h on social media daily (35.0%). The mean age of the participants was 24.0 years (SD: 9.4).

**Table 1:** Demographic characteristics.

Characteristics	Subjects
Age, year, Mean (S.D.)	24.0 (9.4)
Sex, *n* (%)	
Female	778 (54.6)
Male	646 (45.4)
Year of Study, *n* (%)	
First Year	588 (41.3)
Second Year	434 (30.5)
Third Year	223 (15.7)
Fourth Year	179 (12.6)
Marital Status, *n* (%)	
Single	1,214 (85.3)
Married	209 (14.7)
PHQ9 Severity, *n* (%)	
Mild	276 (19.4)
Moderate	98 (6.9)
Moderately Severe	569 (40.0)
Severe	481 (33.8)
GAD7 Severity, *n* (%)	
Minimal	51 (3.6)
Mild	46 (3.2)
Moderate	849 (59.6)
Severe	478 (33.6)
Psychosis, *n* (%)	
No	967 (67.9)
Yes	457 (32.1)
All Mental Health Conditions	
No	984 (69.1)
Yes	440 (30.9)
Financial Support, *n* (%)	
Family	554 (38.9%)
Personal	322 (22.6%)
Loan	229 (16.1%)
Scholarship	306 (21.5%)
Other	13 (0.9%)
Family Support on Academic Life, *n* (%)	
Not supportive	308 (21.6)
Slightly supportive	182 (12.8)
Moderately Supportive	291 (20.4)
Supportive	435 (30.6)
Very Supportive	208 (14.6)
Household Head, *n* (%)	
Yes	83 (26.1)
No	1,052 (73.9)
Utilization of mental health services provided by the university, *n* (%)	
Yes	133 (9.3)
No	1,291 (90.7)
Living Arrangement/Accommodation Status	
On-Campus Halls	197 (13.8)
Off-Campus Private Apartments	1,216 (85.4)
Living at home	11 (0.8)
Insurance for Mental Health, *n* (%)	
No	1,265 (88.8)
Yes	159 (11.2)
Time spent on social media per day (hrs.), *n* (%)	
<1 h	275 (19.3)
1–3 h	498 (35.0)
3–5 h	478 (33.6)
>5 h	173 (12.2)
Awareness of the mental health services available at university, *n* (%)	
No	1,038 (72.9)
Yes	386 (27.1)

### Prevalence of mental health conditions

Table 2 shows the overall prevalence of mental health conditions at 30.9%. This rate is highest among fourth-year students (44.7%), those aged 25–29 years (40.5%), uninsured participants (31.8%), and those living off-campus (33.1%).

**Table 2:** Prevalence of mental health conditions among students.

Variable	Mental Health Condition (*n*, %)		*χ* ^2^	*p*-value
	Yes	No		
Mental Health Condition	440 (30.9)	984 (69.1)		
Family Support on Academic Life				
Not supportive	155 (35.2)	153 (15.6)	94.91	< 0.001
Slightly supportive	37 (8.4)	145 (14.7)		
Moderately Supportive	75 (17.1)	216 (22.0)		
Supportive	91 (20.9)	344 (35.0)		
Very Supportive	82 (18.6)	126 (12.8)		
Utilization of mental health services provided by the university				
Yes	52 (11.82)	81 (8.23)	4.62	0.032
No	388 (88.18)	903 (91.77)		
Year of Study				
First Year	179 (40.68)	409 (41.57)	24.38	< 0.001
Second Year	132 (30.00)	302 (30.69)		
Third Year	49 (11.14)	174 (17.68)		
Fourth Year	80 (18.18)	99 (10.06)		
Living Arrangement/Accommodation Status				
Living at home	3 (0.68)	8 (0.81)	18.64	< 0.001
Off-Campus Private Apartments	402 (91.36)	814 (82.72)		
On-Campus Halls	35 (7.95)	162 (16.46)		
Age of Student				
<20 years	98 (22.27)	372 (37.80)	34.82	< 0.001
20–24 years	272 (61.82)	506 (51.42)		
25–29 years	47 (10.68)	69 (7.01)		
>30 years	23 (5.23)	37 (3.76)		
Insurance for Mental Health				
No	402 (91.36)	863 (87.70)	4.11	0.043
Yes	38 (8.64)	121 (12.30)		
Time spent on social media per day (hrs.)				
< 1 h	119 (27.05)	156 (15.85)	66.88	<0.001
1–3 h	111 (25.23)	387 (39.33)		
3–5 h |	126 (28.64)	352 (35.77)		
>5 h	84 (19.09)	89 (9.04)		
Awareness of the mental health services available at university				
No	312 (70.91)	726 (73.78)	1.268	0.260
Yes	128 (29.09)	258 (26.22)		

### Factors associated with mental health conditions

Participants with highly supportive families had 53% lower odds of developing a mental health condition (AOR: 0.47, 95% CI: 0.29–0.76). Fourth-year students faced a 6% higher risk compared to first-year students (AOR: 1.06, 95% CI: 0.72–1.57), whereas third-year students had 38% lower odds (AOR: 0.62, 95% CI: 0.40–0.97). Increased time spent on social media correlated with a higher risk of mental health conditions. Those spending more than 5 h daily on social media were 2.43 times more likely to develop a mental health condition (AOR: 2.43, 95% CI: 1.47–4.01) (Table 3).

**Table 3:** Factors associated with mental health conditions among students.

	Having Mental Condition (%)	OR (95% CI)	*p*-value	AOR (95% CI)	*p*-value
Family Support on Academic Life					
Not supportive	155 (50.32)	Ref	Ref	Ref	Ref
Slightly supportive	37 (20.33)	0.25 (0.16, 0.39)	<0.001	0.23 (0.13,0.40)	<0.001
Moderately Supportive	75 (25.77)	0.34 (0.24, 0.48)	<0.001	0.27 (0.16, 0.44)	<0.001
Supportive	91 (20.92)	0.26 (0.19, 0.36)	<0.001	0.25 (0.15, 0.39)	<0.001
Very Supportive	82 (39.42)	0.64 (0.45, 0.92)	0.015	0.47 (0.29,0.76)	0.002
Utilization of mental health services provided by the university					
Yes	52 (39.10)	Ref	Ref	Ref	Ref
No	388 (30.05)	0.66 (0.46, 0.97)	0.032	0.74 (0.48, 1.14)	0.177
Year of Study					
First Year	179 (30.44)	Ref	Ref	Ref	Ref
Second Year	132 (30.41)	0.99 (0.76, 1.31)	0.992	1.02 (0.72, 1.45)	0.902
Third Year	49 (21.97)	0.64 (0.45,0.92)	0.017	0.62 (0.40, 0.97)	0.037
Fourth Year	80 (44.69)	1.85 (1.31, 2.60)	<0.001	1.06 (0.72, 1.57)	0.757
Living Arrangement/Accommodation Status					
On-Campus Halls	35 (17.77)	Ref	Ref	Ref	Ref
Off-Campus Private Apartments	402 (33.06)	2.28 (1.55, 3.36)	<0.001	1.52 (1.0, 2.30)	0.048
Living at home	3 (27.27)	1.73 (0.43, 6.87)	0.432	1.2 (0.28, 5.25)	0.803
Age of Student					
<20 years	98 (20.85)	Ref	Ref	Ref	Ref
20–24 years	272 (34.96)	2.0 (1.56, 2.66)	<0.001	1.78 (1.27, 2.49)	0.001
25–29 years	47 (40.52)	2.6 (1.67, 3.98)	<0.001	1.42 (0.86, 2.35)	0.168
>30 years	23 (38.33)	2.4 (1.33, 4.16)	0.003	1.11(0.59, 2.09)	0.748
Insurance for Mental Health					
No	402 (31.78)	Ref	Ref	Ref	Ref
Yes	38 (23.90)	0.67 (0.45,0.99)	0.044	0.61 (0.39,0.96)	0.031
Time spent on social media per day (hrs.)					
< 1 h	119 (43.27)	Ref	Ref	Ref	Ref
1–3 h	111 (22.29)	0.37 (0.27,0.52)	<0.001	0.89(0.59, 1.33)	0.577
3–5 h |	126 (26.36)	0.46 (0.34,0.64)	<0.001	1.18(0.77, 1.82)	0.424
>5 h	84 (48.55)	1.23 (0.84, 1.81)	0.274	2.43 (1.47, 4.01)	<0.001
Awareness of the mental health services available at university					
No	312 (30.06)	Ref	Ref	Ref	Ref
Yes	128 (33.16)	1.15 (0.89, 1.48)	0.260	1.38 (1.01, 1.87)	0.040

## Discussion

Students who perceived their families as unsupportive had the highest prevalence of mental health conditions (35.2%). This finding aligns with psychological and public health research, which suggests that family support plays a crucial role in mental wellbeing (32, 33). According to Social Support Theory, strong family bonds provide emotional security, reduce stress, and promote resilience against mental health challenges (34). Conversely, a lack of support can lead to feelings of isolation, increased stress, and a higher risk of anxiety and depression. Additionally, Attachment Theory suggests that early family relationships shape emotional regulation and coping mechanisms, influencing mental health outcomes in adulthood (35, 36). These findings highlight the critical role of a supportive family environment in protecting students from mental health conditions (33).

According to Yang et al. (32), strong family support enhances coping mechanisms and reduces the risk of mental health issues among students. These findings emphasize the importance of a supportive family environment in protecting students from mental health challenges. Policymakers and schools should consider integrating family engagement initiatives to strengthen family cohesion as part of mental health interventions.

Students who did not access university mental health services had a higher prevalence of mental health conditions (88.2%). However, they had 34% lower odds of experiencing a mental health condition. This finding suggests that students with mental health issues are more likely to seek university mental health services or that barriers may exist in effectively utilizing these services. The findings align with existing literature, indicating that students with severe mental health concerns are more likely to seek professional help, while those relying on alternative coping mechanisms may avoid mental health services (37). The observed associations may be explained by several mechanisms. First, students who experience severe mental health conditions may have a higher likelihood of seeking university mental health services due to the intensity of their symptoms and the need for professional support. Conversely, students who did not access these services but reported a high prevalence of mental health conditions may rely on alternative coping strategies, such as social support networks, self-care, or informal counseling, which could contribute to lower odds of being diagnosed or self-reporting a mental health condition. Another study found that stigma and cultural attitudes are significant barriers to university students using mental health services (38). Universities should focus on removing these obstacles to ensure all students have equal access to support and can seek help early.

First-year students reported the highest rates of mental health conditions (40.7%), likely due to the challenges of adjusting to university life, including academic pressure, social transitions, and increased independence (39, 40). In contrast, third-year students had significantly lower odds, possibly because of improved coping mechanisms, stronger social networks, and greater familiarity with academic demands (41). However, fourth-year students exhibited an increased risk, potentially due to the stress associated with academic completion, career uncertainty, and the transition to the workforce. These findings highlight the need for universities to implement targeted mental health interventions, such as transition support for first-year students, resilience programs for mid-level students, and career counseling for final-year students to promote the overall wellbeing (39).

The higher prevalence of mental health conditions among off-campus students (91.4%) and their 2.3 times greater odds highlight the critical role of accommodation in student wellbeing. Beyond statistical significance, these findings reflect real-life challenges such as social isolation, financial stress, and reduced access to university support. On-campus housing provides structured support, social connections, and easier access to mental health resources, whereas off-campus students may face loneliness and difficulty balancing responsibilities (41). Universities should recognize housing as a key factor in student mental health and implement strategies such as extending campus-based support, fostering virtual peer networks, and providing financial aid to ensure the wellbeing of all students (42).

The significant influence of age on student mental health, with 61.8% of those aged 20–24 years reporting challenges, reflects stress related to academic pressures, independence, and life transitions. This finding aligns with the Healthy Minds Study (2023), which found that over 60% of college students met the criteria for at least one mental health condition (43, 44). Beyond statistics, these findings highlight the need for targeted mental health support that addresses factors such as social expectations, financial stress, and digital influences. Universities should implement proactive strategies, including accessible counseling, peer support, and stress management programs, to support students during this critical developmental stage (45).

Students aged 25–29 years were 2.6 times more likely to experience mental health conditions than those under 20 years, likely due to career uncertainties, academic pressure, and financial instability. This elevated risk underscores the growing mental health challenges faced by older students as they navigate complex life transitions. The 2024 Healthy Minds Network reported that 61% of college students with anxiety or depression sought counseling services, reflecting both the increasing demand for support and a positive shift in help-seeking behavior (44). These findings highlight the need for universities to expand targeted mental health interventions, particularly for older students balancing academics with personal and financial responsibilities.

Students without mental health insurance were significantly more likely to screen positive for mental health conditions, with 91.4% testing positive, while those with insurance were more likely to face a 33% lower risk of testing positive. These findings align with a 2020 study showing that health insurance enrollment enhances access to mental health care (46). However, several factors may influence this association. Socioeconomic status could play a role, as students from wealthier backgrounds may afford insurance and access private mental health services. Additionally, insured students may be more proactive in seeking care, have better access to university support services, and experience fewer financial stressors. On the other hand, uninsured students may face greater academic and work-related pressures, further increasing their risk. These findings highlight the need to address disparities in access to mental health care, particularly for uninsured students, to improve overall wellbeing.

The study found a significant association between social media use and mental health conditions, with students spending 3–5 h per day having the highest prevalence (28.6%), while those using 1–3 h daily had a 63% reduced risk. These findings align with a 2021 study showing that moderate use enhances peer interactions, but excessive use increases stress and mental health risks (47). Clinically, excessive social media use may contribute to anxiety, depression, and sleep disturbances by disrupting circadian rhythms and exposing users to harmful content (47). To mitigate these risks, universities and health care providers should integrate social media usage assessments into mental health screenings, promote digital wellbeing programs, and encourage mindful social media engagement to support student mental health.

The study found that awareness of university mental health services does not significantly correlate with mental health outcomes, as 70.9% of students who were aware of these services still screened positive for mental health conditions. This finding aligns with a 2022 systematic review, which highlighted that, while awareness is important, it does not necessarily lead to service utilization or improved outcomes among university students. Beyond statistical significance, these findings suggest that barriers such as stigma, perceived ineffectiveness of services, long wait times, and personal reluctance to seek help may limit the impact of awareness alone (37). To improve mental health outcomes, universities should focus on reducing stigma, enhancing service accessibility, and actively promoting engagement with available mental health resources.

## Conclusion

This study provides valuable insights into the factors influencing students’ mental health, highlighting the critical role of family support, mental health insurance, and social media usage. By identifying first-year and final-year students as particularly vulnerable groups, the findings contribute to a deeper understanding of how academic transitions impact mental wellbeing. These insights have significant implications for universities and policymakers, emphasizing the need for targeted interventions such as family engagement initiatives, improved student housing, and tailored mental health support programs. Strengthening these structural and social support systems can enhance students’ wellbeing during their academic journey and beyond, ultimately fostering a healthier university environment.

## Data Availability

The datasets presented in this study can be found in online repositories. The names of the repository/repositories and accession number(s) can be found by searching INSPIRE Mental health data Kilifi here: https://aphrc.org/microdata-portal/.(48)
